# Task Travel Time Prediction Method Based on IMA-SURBF for Task Dispatching of Heterogeneous AGV System

**DOI:** 10.3390/biomimetics10080500

**Published:** 2025-08-01

**Authors:** Jingjing Zhai, Xing Wu, Qiang Fu, Ya Hu, Peihuang Lou, Haining Xiao

**Affiliations:** 1 College of Mechanical and Electrical Engineering, Nanjing University of Aeronautics and Astronautics, Yudao Street, Nanjing 210016, China; zhaijingjing@nuaa.edu.cn (J.Z.); huyanuaa@163.com (Y.H.); meephlou@nuaa.edu.cn (P.L.); 2 State Key Laboratory of Intelligent Manufacturing System Technology, Beijing Institute of Electronic System Engineering, Beijing 100854, China; qiang_fox@buaa.edu.cn; 3 College of Mechanical Engineering, Yancheng Institute of Technology, Hope Avenue, Yancheng 224051, China; xiao226563@163.com

**Keywords:** automatic guided vehicle, heterogeneous system, task travel time prediction, radial basis function, mayfly algorithm

## Abstract

The heterogeneous automatic guided vehicle (AGV) system, composed of several AGVs with different load capability and handling function, has good flexibility and agility to operational requirements. Accurate task travel time prediction (T3P) is vital for the efficient operation of heterogeneous AGV systems. However, T3P remains a challenging problem due to individual task correlations and dynamic changes in model input/output dimensions. To address these challenges, a biomimetics-inspired learning framework based on a radial basis function (RBF) neural network with an improved mayfly algorithm and a selective update strategy (IMA-SURBF) is proposed. Firstly, a T3P model is constructed by using travel-influencing factors as input and task travel time as output of the RBF neural network, where the input/output dimension is determined dynamically. Secondly, the improved mayfly algorithm (IMA), a biomimetic metaheuristic method, is adopted to optimize the initial parameters of the RBF neural network, while a selective update strategy is designed for parameter updates. Finally, simulation experiments on model design, parameter initialization, and comparison with deep learning-based models are conducted in a complex assembly line scenario to validate the accuracy and efficiency of the proposed method.

## 1. Introduction

### 1.1. Industrial Requirements

The automated guided vehicle (AGV) has emerged as a key logistics delivery robot, integral to various manufacturing, warehousing, and logistics systems, and playing a pivotal role in the ongoing trend of industrial automation [1,2,3]. Traditional AGV systems, typically consisting of a single type of vehicle, face significant limitations in terms of material transport capabilities, which restricts their applicability, especially in small-batch, multi-variety production environments. These limitations often result in inefficiencies and underutilization, particularly in modern smart manufacturing scenarios with diversified production demands. In response to these challenges, heterogeneous AGV systems—comprising multiple types of AGVs with varying load capacities and handling functionalities—have gained prominence in industrial applications [4,5]. These systems include single-load AGVs, which carry one task at a time, and multi-load AGVs, capable of carrying multiple tasks simultaneously to improve handling efficiency [6]. The flexibility of heterogeneous AGV systems enables them to adapt more effectively to fluctuating production demands and dynamic task requirements, thus fostering the development of intelligent manufacturing solutions tailored to meet the specific needs of customers [7].

Optimizing the performance of heterogeneous AGV systems relies on three key enabling technologies: task scheduling, path planning, and traffic management [8,9,10]. Among these, accurate task travel time prediction (T3P) is essential for improving dispatching and resource utilization. To this end, a biomimetics-inspired learning framework based on a radial basis function (RBF) neural network with an improved mayfly algorithm and a selective update strategy (IMA-SURBF) is proposed. The improved mayfly algorithm (IMA) draws on the biological principles of the flight behavior and mating process of mayflies, where these biomimetic mechanisms are employed to initialize the parameters of the RBF neural network. By introducing this biomimetics-inspired initialization mechanism, the framework effectively captures complex task correlations and adapts to dynamic model structures, thereby significantly enhancing prediction accuracy.

### 1.2. Literature Review

However, T3P is complicated due to the relevance and diversity of tasks, so an advanced method is required for the T3P. In addressing the T3P problem, the existing research work primarily focuses on two major transportation fields: urban roads and highways. The commonly used T3P methods are classified into traditional and machine learning methods [11,12,13].

#### 1.2.1. Traditional T3P Method

T3P involves numerous factors including historical data, traffic conditions, and real-time information, requiring context-appropriate methods often combining multiple techniques. Traditional methods include statistical, time series, and regression analyses [14,15,16]. Although limited under unstable traffic or complex settings, they provide valuable insights for specific cases [17]. For example, Woodard et al. [18] introduced a method to predict the probability distribution of travel time on an arbitrary route in a road network at an arbitrary time, using GPS data from mobile phones or other probe vehicles, and this method was the first method to provide accurate predictions of travel time reliability for complete, large-scale road networks. In addition to GPS data, Xinghao et al. [19] proposed a bus travel time prediction model considering delay caused by signal control and the acceleration and deceleration by combining GPS data and RFID data; application of the model after the equipment of RFID devices would significantly improve the accuracy of travel time prediction. Among the traditional methods, regression analysis was the most popular one. Osei et al. [20] and Ming et al. [21] used multiple linear regression model and a novel Burr mixed autoregressive model, respectively, to predict travel time that would capture the contributing factors of congestion typical of low-income country arterial road environment and flow characteristics, and for the intermediate-to-long term period of bus section travel time prediction, providing reference for bus line operation and scheduling planning. In addition to taxis and buses, Qi et al. [22] also presented a discrete and continuous combined analysis for attaining improved long-term travel time prediction of commercial vehicles, which could be tactically helpful in predicting long-term travel time ahead of the scheduled trips to improve the reliability of the schedules.

However, due to the limitations of data acquisition and data distribution, it is difficult to implement the above-mentioned traditional methods. Contrarily, the mathematical modeling-based methods are widely used in scenarios with limited data. By analyzing the important factors affecting travel time, a suitable mathematical model was constructed to explore the potential relationship between travel time and influencing factors [23]. For example, Davis [24] proposed a new method to predict the travel time on a highway route with a bottleneck caused by an on-ramp. It used the advantage of the slow variation of the bottleneck throughput in the existence of congestion. The simulation results showed that the travel time converged to the target value, and remained close to or below it by using the proposed prediction strategy. Bharathi et al. [25] explored the suitability of higher-order traffic flow models for the prediction of bus travel time to addressed most of the limitations of the previous models. In order to capture the random variations in travel time, Dhivyabharathi et al. [26] used a dynamic mathematical modeling approach with particle filtering technique. The performance of this method was better than the method based on the time–distance relationship of space mean speed. Koh et al. [27] studied the characteristics of a warehousing system, in which the storage and retrieval orders were performed by a tower crane. A mathematical travel time model was developed, including the derivation of the single command cycle and the double command cycle under the random storage allocation rule. The travel time was estimated based on the turnover-based assignment rule by using a numerical approach.

#### 1.2.2. Machine Learning-Based T3P Method

In addition to the aforementioned traditional methods, machine learning methods such as decision tree, support vector machine, random forest, K-nearest neighbor algorithm, naive Bayes, and neural network have become important tools for T3P [28,29]. For example, Sakhare and Vanajakshi [30] used linear regression and artificial neural network (ANN) technology to establish models, respectively, and used bus travel time obtained from GPS to estimate stream travel time. The experimental results showed that ANN performed better compared with the linear regression for all sizes of segments. Shao et al. [31] developed a machine learning-based generative model, which used license plate recognition data for travel time distribution prediction. Serin et al. [32] used machine learning methods with three-layer architecture to predict bus arrival time. The experimental results showed that radial basis function (RBF) neural network had good prediction performance and the three-layer architecture provided successful results with approximately 2.552 MAPE.

Due to the inherent limitations of standalone machine learning methods, many studies have attempted to enhance prediction accuracy by combining multiple machine learning techniques or integrating them with nature-inspired metaheuristic algorithms [33,34,35].For example, a recent comprehensive survey by Ghiaskar et al. (2024) [36] provides an in-depth review of the latest developments in nature-inspired optimization algorithms, highlighting their increasing application in hybrid learning frameworks, continually being proposed to further enhance performance. Lin et al. [33] presented an innovative methodological framework that integrated the exponential smoothing technique, artificial neural network, and Bayes algorithms for predicting the travel time along a signalized corridor. The testing experiment in real-world travel time datasets indicated a good performance of the travel time prediction models. Nimpanomprasert et al. [34] combined multilayer perceptrons and a long short-term memory neural network with genetic algorithm to predict the bus travel time on a given time of day, a given day of week, and under a given weather condition. The experimental results showed that the hybrid model could effectively predict the bus travel time. Zhao et al. [35] proposed a Bayesian encoder–decoder deep neural network model to predict bus travel time, which was organically combined with a visualization model to better demonstrate the prediction results and uncertainties for user perception and decision-making.

### 1.3. Research Gap and Our Contribution

The comparative analysis of T3P literature is shown in Table 1. We can find that there is still a gap between the research work into the problem of task travel time and the dispatching application of heterogeneous AGV systems.

(1) Travel-influencing factors are different. The T3P accuracy of the heterogeneous AGV system is affected by the specific factors of the AGV task, such as loading and unloading points, guidance paths, etc. Moreover, there is a correlation among several sub-tasks of the multi-load AGV task in the heterogeneous AGV system, such as the loading and unloading sequence of the sub-task, the path coupling of different sub-tasks, etc. The heterogeneous AGV system can obtain the detailed influencing factors for each individual task, which is conducive to improving the T3P accuracy. However, it is difficult to obtain such detailed influencing factors for traffic tasks in the transportation field. Only some macro-influencing factors are considered for traffic tasks, such as the number of vehicles, weather, date, and other factors. Therefore, the existing T3P models in the transportation field are not fully applicable to the prediction problem of the heterogeneous AGV system.

(2) The input/output vector dimensions of the prediction model are different. The travel-influencing factors are used as the input vector of the prediction model. On the one hand, because of the dynamic change of the number of AGV tasks in a complex assembly line, the input/output dimension of the prediction model for the heterogeneous AGV system also changes. It brings great difficulties to the design and update of the prediction model. On the other hand, the number of macro-influencing factors considered in the transportation field is fixed, so the input/output dimension of the prediction model for traffic tasks can be determined in advance. It meets the requirements for the input/output dimension of general prediction model. Therefore, the existing prediction modeling method in the transportation field is not suitable for the model with dynamic input/output dimensions needed in the heterogeneous AGV system.

Therefore, unlike the T3P problem in the transportation field, this paper, for the first time, presents the T3P problem in heterogeneous AGV systems, which is characterized by task correlation and dynamic changes in model input/output dimensions. To address these challenges, a T3P method based on IMA-SURBF is proposed. The main contributions of this study are as follows.

(1) Innovative model design method. Based on the structure of RBF neural network, travel-influencing factors related to traffic flow, handling task, and AGV configuration are taken as the input, and the task travel time of handling tasks is taken as the output. The dynamic input/output strategy is used to solve the problem of dynamic changes in the input/output dimension of prediction model. (2) Improved model updating method. The improved mayfly algorithm is developed with two improvement points to optimize the initial weights of RBF neural network, which makes up for the deficiency that traditional RBF is inclined to fall into the local optimum. Furthermore, a selective update strategy is designed to prevent invalid data in the sample input matrix from degrading training speed and prediction accuracy.

The remainder of the paper is organized as follows. Section 2 describes the problem and the general scheme of AGV dispatching with T3P. Section 3 introduces the T3P method based on IMA-SURBF. The simulation experiments are carried out and the experimental results are analyzed in Section 4. Finally, conclusions and future research work are given in Section 5.

## 2. General Scheme

### 2.1. Problem Description

**Figure 1:**
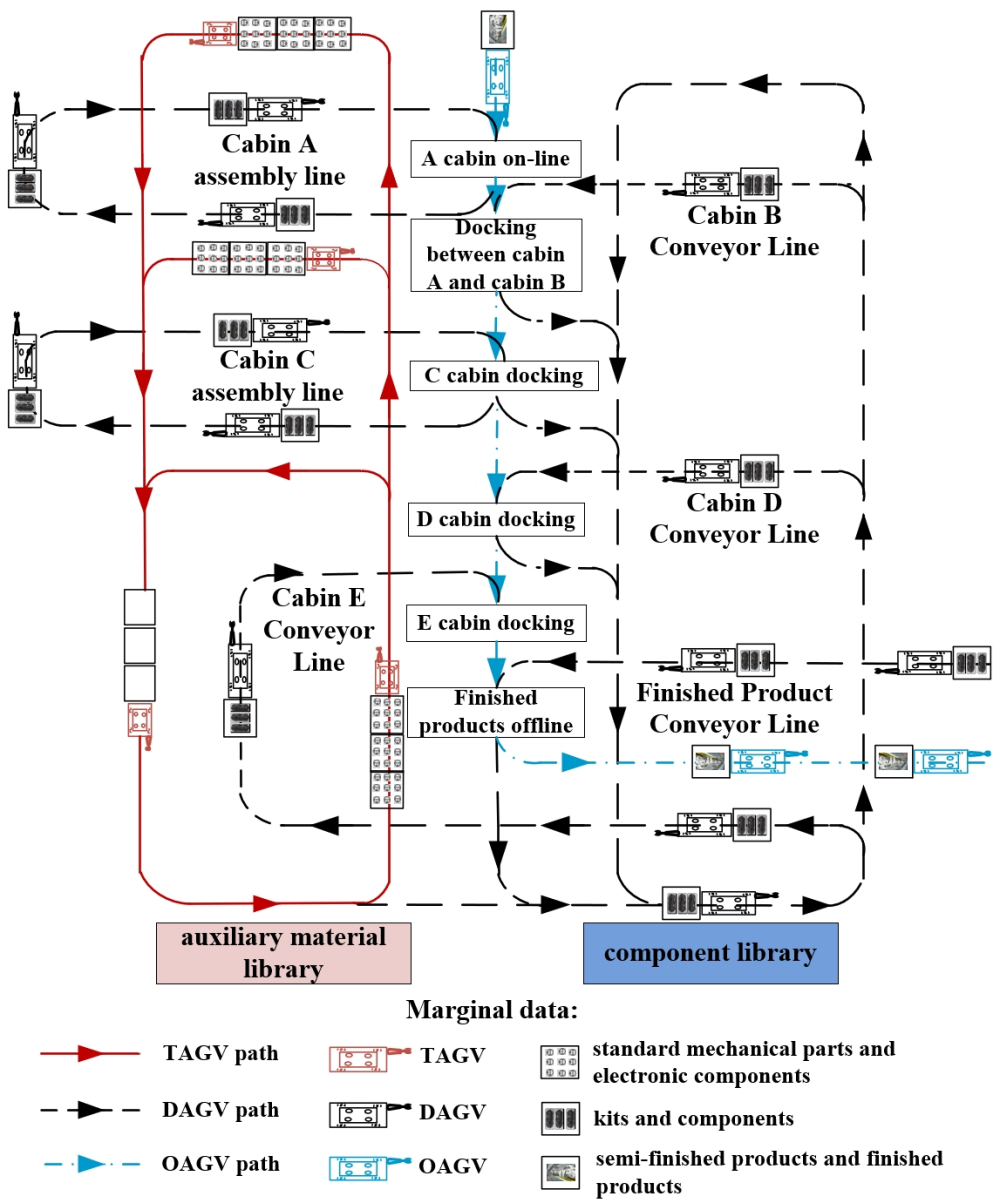
Assembly line logistics system.

(1) Description of heterogeneous AGV system. Currently, the AGV system has been widely used in the manufacturing assembly and logistics field, significantly enhancing the system efficiency and resource utilization. Compared with the homogeneous AGV system containing one kind of AGVs, a heterogeneous AGV system offers greater flexibility, stability, and scalability. Moreover, it is excellent for scenarios with a wide variety of materials and different task requirements. In the above scenarios, it is difficult to predict the task travel time because it is affected by many influencing factors. However, accurate T3P is crucial for achieving the efficient operation of heterogeneous AGV systems.

(2) Example Description. Taking a typical complex assembly line as an example, it consists of three assembly lines of cabin A assembly line, cabin C assembly line, and cabin final assembly line, and three conveyor lines of cabin B, cabin D, and cabin E, as shown in Figure 1. Further, a unidirectional guidance path network is adopted for logistics transport. The assembly line is characterized by a wide variety of materials and different task requirements, and there are problems such as long turnaround time for semi-finished products and poor distribution punctuality. To solve the above problems, we design a heterogeneous AGV system as its material handling system. Specifically, the multi-load trailer AGVs (TAGV) are used to distribute standard mechanical parts and electronic components. The single-load differential AGVs (DAGV) are used to distribute the kits and components, and the single-load omnidirectional AGVs (OAGV) are used to distribute the semi-finished products and finished products.

(3) Description of task travel time. To ensure the on-time delivery of assembly materials to each assembly station, a reasonable task-dispatching scheme is needed for the efficient and orderly operation of assembly lines. Task delay time or task completion time are two important indicators when solving a task-dispatching problem. Both are affected by task start time and task travel time. While the task start time is known at the time of dispatching, the task travel time is usually uncertain. In order to solve the task-dispatching problem better, it is necessary to design an effective method for predicting the task travel time.

### 2.2. General Scheme

**Figure 2:**
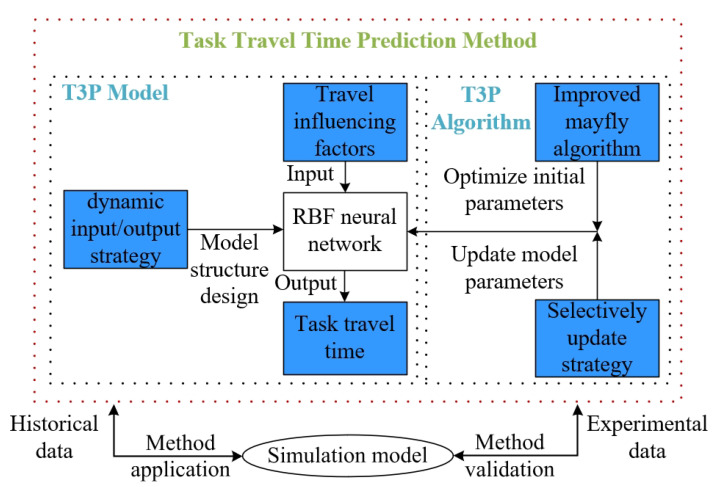
General scheme.

In order to solve the problem that the task travel time of a heterogeneous AGV system is difficult to predict, this paper proposes a T3P method based on IMA-SURBF. As shown in Figure 2, the blue background in the figure is the key content to be studied in this paper.

According to the characteristics of the T3P problem of a heterogeneous AGV system, the T3P model is constructed by taking the travel-influencing factors as input and the task travel time as output. These influencing factors are related to distribution task, AGV individual and traffic flow, etc. The input/output dimension of RBF neural network is determined by a dynamic input/output strategy. Then, aiming at the defects of traditional RBF neural network, a T3P algorithm based on IMA and a selective update strategy is proposed. Finally, the performance experiment is conducted for the T3P algorithm.

## 3. Task Travel Time Prediction

### 3.1. T3P Model

The commonly-used neural networks include the back propagation (BP) neural network and the RBF neural network. It has been proved that the RBF neural network has stronger and faster nonlinear fitting ability than the BP neural network [37]. The RBF neural network offers many advantages, such as simple structure, fast convergence speed, and the ability to approximate any nonlinear function [38]. Therefore, this paper constructs a T3P model based on the RBF neural network. While neural networks, particularly RBF networks, have been extensively applied in task prediction, their application to heterogeneous AGV systems has been limited. Heterogeneous AGV systems, characterized by diverse vehicle types, variable task demands, and dynamic traffic flow environments, present unique challenges for T3P. The novelty of this study lies in the development of the T3P model, which combines the characteristics of heterogeneous AGV systems with RBF neural networks to address these challenges. The proposed model dynamically adapts to changes in task assignments, traffic flow, and AGV states, improving prediction accuracy in heterogeneous AGV systems.

Based on the network structure of a typical RBF neural network, the topology structure of the T3P model is further determined according to the characteristics of a heterogeneous AGV system and the requirements of the problem in this paper. The input layer X, hidden layer Z, and output layer Y of the network can be represented by the following matrix:

(1) $$\begin{array}{c} X={\left(x_{1},x_{2},\ldots ,x_{n}\right)}^{T}\\ Z={\left(z_{1},z_{2},\ldots ,z_{p}\right)}^{T}\\ Y={\left(y_{1},y_{2},\ldots ,y_{q}\right)}^{T} \end{array}$$

where n,p,q denote the number of neurons in the input layer, hidden layer, and output layer, respectively. Based on the heuristic guidelines summarized in [39], the number of hidden nodes *p* is determined as p=1.5×n.

**Figure 3:**
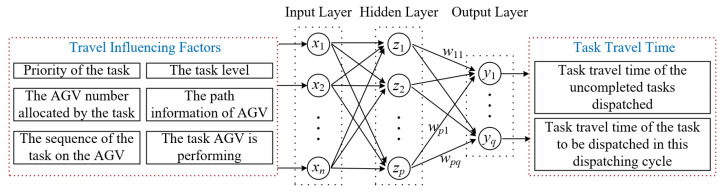
Topology structure of the T3P model.

The topology structure of the T3P model proposed in this paper is shown in Figure 3. In order to ensure the certainty of the model topology, a dynamic input/output strategy is used to determine *n* and *q*. Specifically, to X, the total number of elements *n* is the product of the maximum number $N_{n}$ of tasks possible in the system and the number *l* of travel-influencing factors. To Y, the size of q is equal to $N_{n}$ of tasks in the system.

The input layer X represents the tasks, the traffic load of paths, and the load of the AGVs in the system at a given time. In X, the set of every *l* elements from the front to the back represents travel-influencing factors of a task. These factors include the priority of the task, the AGV number assigned by the task, the sequence of the task on the AGV, the path information of AGV, the task that AGV is performing, and the task level (the uncompleted tasks that have been dispatched or the tasks to be dispatched in this dispatching cycle). Moreover, considering the dimensional influence between the travel-influencing factors, the input matrix X must be normalized before being put into the RBF network for parameter update. The output layer Y represents the task travel time in the system. Tasks include the uncompleted tasks dispatched or the task to be dispatched in this dispatching cycle.

### 3.2. T3P Algorithm

The conventional RBF neural network does not introduce additional parameters in the whole training process. It only adjusts the initial weight, the centers and widths of the basis functions, and deviations according to the training samples. Consequently, it is prone to fall into the local optima. Moreover, the prediction performance of the RBF neural network is affected by the parameters of the weight, and the center and width of the hidden layer. When the initial parameters are randomly selected, the solution process tends to fall into a local extreme point, leading to deviation from the optimal parameters and a low-performance network model. Furthermore, the dimensions of the input layer, the output layer, and the weight parameters in the conventional RBF neural network are all fixed on the updating stage during the network training process. In contrast, the distribution tasks in the AGV system are updated in real time and the task number changes dynamically. If the input/output variables of the RBF neural network are set according to the real-time data in the system, the dimensions of variables and parameters in the RBF neural network will change irregularly. It makes the T3P model too complicated to solve. To address these challenges, this study proposes the following enhancements to the traditional RBF neural network.

(1) K-means algorithm is used to determine the center and width of the basis functions, and an IMA is developed to optimize the initial weights of the RBF neural network. On the one hand, the K-means method is simple and easy to implement. It can reduce the computational load of the RBF neural network algorithm while approaching to the optimal value as close as possible [40]. In our model, the K-means algorithm is used to determine the center and width of the basis functions in the RBF neural network. Specifically, K-means is applied to cluster the input data, where the centroids of the clusters are used as the centers of the radial basis functions, and the spread of each cluster is used to define the width of the corresponding basis function. On the other hand, the mayfly algorithm (MA) is a new intelligent optimization algorithm with advantages in terms of convergence rate and speed [41,42]. However, the conventional MA is prone to fall into the local optima in the later stage. Therefore, The IMA algorithm, an enhanced variant of the standard mayfly algorithm, is utilized to optimize the parameters of the RBF neural network for better prediction accuracy.

(2) A selective updating strategy is designed by dynamically selecting partial weight parameters to update. The model topology is fixed, while the dimensions of input/output variables and internal parameters change under the upper limitation, which is determined by the maximum number of tasks possible in the AGV system. The practical task number in the AGV system varies at different moments. If the practical task number is smaller than the upper limitation, some remaining (the upper limitation minus the practical task number) rows of the input matrix are all meaningless or invalid data. Since the invalid data cannot describe any system characteristics, updating the weight parameters according to the invalid data during the training process not only wastes training time but also brings negative effects to the model accuracy. Hence, the selective update strategy is designed to select the valid data in the input rows for parameter training while disregarding the invalid data in the remaining rows. The strategy promises to improve the training efficiency and prediction accuracy of the RBF neural network simultaneously.

**Figure 4:**
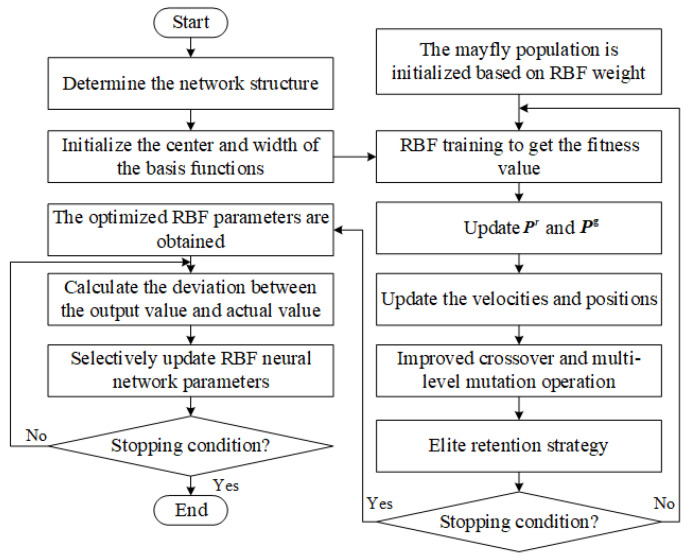
The IMA-SURBF flowchart.

The flowchart of the IMA-SURBF algorithm is shown in Figure 4. The detailed algorithm steps are as follows.

Step1: Initialize RBF neural network parameters. To accurately reflect the sample’s real situation and ensure the precision of the prediction model, this paper uses the K-means algorithm to initialize the center $C_{j}={\left(c_{j1},c_{j2},\ldots ,c_{jn}\right)}^{T}$ and width $D_{j}\left(j=1,2,\ldots ,p\right)$ of the basis function.

Step2: Determine the initial weight $W_{k}={\left(w_{k1},w_{k2},\ldots ,w_{kp}\right)}^{T}\left(k=1,2,\ldots ,q\right)$.

Step2.1: Initialize the mayfly population. To obtain the solution space of the weight, each mayfly individual’s position is used to represent a weight solution. Further, the dimension D=p∗q of the weight denotes the dimension of the mayfly individual vector. Then, the size of male and female populations is set to $N_{MA}$, and the serial number of each mayfly individual is marked as $r\left(r=1,2,\ldots ,N_{MA}\right)$. The position $x^{r}=\left(x_{1}^{r},x_{2}^{r},\ldots ,x_{D}^{r}\right)$ and velocity ${\mathrm{vm}}^{r}=\left(vm_{1}^{r},vm_{2}^{r},\ldots ,vm_{D}^{r}\right)$ of each male mayfly individual are initialized. Similarly, the position $y^{r}=\left(y_{1}^{r},y_{2}^{r},\ldots ,y_{D}^{r}\right)$ and velocity ${\mathrm{vf}}^{r}=\left(vf_{1}^{r},vf_{2}^{r},\ldots ,vf_{D}^{r}\right)$ of each female mayfly individual are also initialized. Furthermore, $p^{r}=\left(p_{1}^{r},p_{2}^{r},\ldots ,p_{D}^{r}\right)$ represents the best position found by the rth mayfly that had ever been visited, and $p^{g}=\left(p_{1}^{g},p_{2}^{g},\ldots ,p_{D}^{g}\right)$ denotes the global best position. Finally, the range of position and speed values, along with the maximum iteration number $E_{0}$, are set.

Step2.2: Calculate the fitness value. For each individual mayfly, it is firstly decoded into the weights of the neural network. Then, a complete neural network is constructed with the weights obtained by decoding, and the center and width are determined using the K-means algorithm. Thirdly, the neural network is trained by using the sample data. Finally, the mean square error (MSE) obtained by network training is used as the fitness value of the individual mayfly.

Step2.3: Update $p^{r}$ and $p^{g}$. The current fitness value of the rth mayfly is compared with its historical optimal fitness value. If the current fitness value is better, the optimal position $p^{r}$ and fitness value of the *r*th mayfly are updated. Then the global optimal mayfly individual and position $p^{g}$ are updated.

Step2.4: Update velocities and positions.

Movement of male mayflies: Assuming $x^{r}\left(s\right)$ is the current position of *r*th mayfly in the search space at time step *s*, the current position is changed by adding a velocity, ${\mathrm{vm}}^{r}\left(s+1\right)$, to the next position. This can be formulated as

(2) $$x^{r}\left(s+1\right)=x^{r}\left(s\right)+{\mathrm{vm}}^{r}\left(s+1\right)$$

When the fitness value of rth mayfly is less than the fitness value of $p^{g}$, the velocity is calculated as

(3) $$vm_{t}^{r}\left(s+1\right)=vm_{t}^{r}\left(s\right)+\alpha_{1}e^{-\beta r_{p}^{2}\left(p_{t}^{r}-x_{t}^{r}\left(s\right)\right)}+\alpha_{2}e^{-\beta r_{g}^{2}\left(p_{t}^{g}-x_{t}^{r}\left(s\right)\right)}$$

where $vm_{t}^{r}\left(s\right)$ is the velocity of *r*th mayfly in dimension t=1,2,…,D at time step *s*, $x_{t}^{r}\left(s\right)$ is the position of *r*th mayfly in dimension *t* at time step *s*, $\alpha_{1}$ and $\alpha_{2}$ are positive attraction constants used to scale the contribution of the cognitive and social component, respectively. β is a fixed visibility coefficient, used to limit a mayfly’s visibility to others, while $r_{p}$ is the Cartesian distance between $x^{r}$ and $p^{r}$, and $r_{g}$ is the Cartesian distance between $x^{r}$ and $p^{g}$. The squared distances $r_{p}^{2}$ and $r_{g}^{2}$ are used inside the exponential functions to model Gaussian-like decay of attraction strength [43].

When the fitness value of rth mayfly is better than the fitness value of $p^{g}$, the velocity is calculated as

(4) $$vm_{t}^{r}\left(s+1\right)=vm_{t}^{r}\left(s\right)+d\gamma$$

where *d* is the nuptial dance coefficient and γ is a random value in the range [−1, 1].

Movement of female mayflies: Assuming $y^{r}\left(s\right)$ is the current position of female mayfly *r* in the search space at time step *s*, the current position is changed by adding a velocity ${\mathrm{vf}}^{r}\left(s+1\right)$ to the next position, i.e.,

(5) $$y^{r}\left(s+1\right)=y^{r}\left(s\right)+{\mathrm{vf}}^{r}\left(s+1\right)$$

The velocity of female mayfly is defined by different equations according to the fitness value of male mayfly. When the fitness value of the *r*th female mayfly is better than that of the *r*th male mayfly, the velocity is defined by Equation (6); otherwise, Equation (7) is used:

(6) $$vf_{t}^{r}\left(s+1\right)=vf_{t}^{r}\left(s\right)+\alpha_{2}e^{-\beta r_{mf}^{2}\left(x_{t}^{r}\left(s\right)-y_{t}^{r}\left(s\right)\right)}$$

(7) $$vf_{t}^{r}\left(s+1\right)=vf_{t}^{r}\left(s\right)+fl\times \gamma$$

where $vf_{t}^{r}\left(s\right)$ is the velocity of *r*th female mayfly in dimension t=1,2,…,D at time step *s*, $y_{t}^{r}\left(s\right)$ is the position of *r*th female mayfly in dimension *t* at time step *s*, $r_{m}f$ is the Cartesian distance between male and female mayflies, and fl is a random walk coefficient.

**Figure 5:**
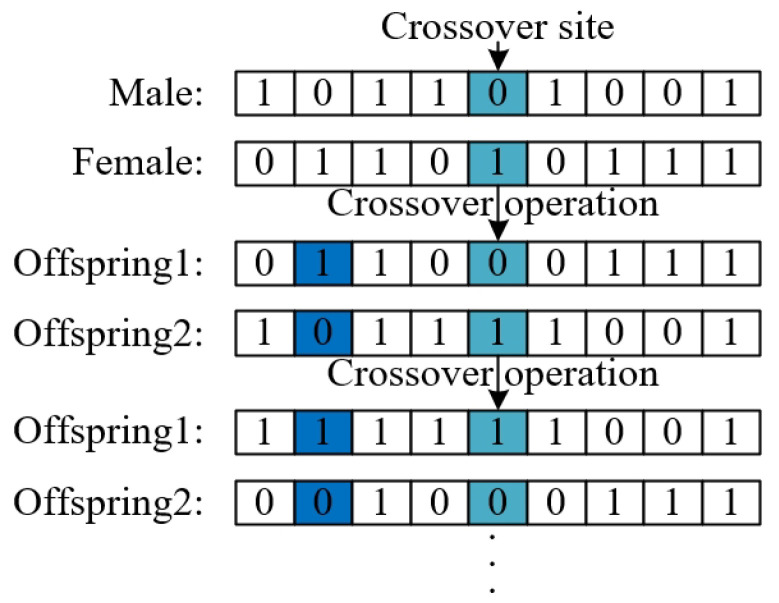
The second crossover operation. Color key: light blue represent the first randomly selected crossover site in Step 7 of Algorithm 1; dark blue represent the second randomly selected crossover site; white represent the remaining site.

Step2.5: Improved crossover operation. The crossover operation according to Equation (8) has the limitation that is not easy to escape local optima. To enhance the population diversity, an improved crossover operation is proposed for the MA. The main steps of improved crossover operation are presented in Algorithm 1, as illustrated in Figure 5.

(8) $$\left\{\begin{array}{cc} \mathrm{offspring}1 & =L\cdot \mathrm{male}+\left(1-L\right)\cdot \mathrm{female}\\ \mathrm{offspring}2 & =L\cdot \mathrm{female}+\left(1-L\right)\cdot \mathrm{male} \end{array}\right.$$

where male is the male parent, female is the female parent, and *L* is a random value within a specific range.
**Algorithm 1** Improved crossover operation**Input:** Male and female populations, Number of offspring num_off, crossover probability $P_{c}$**output:** offspring populations1:**for** i=1 to $\frac{num\_off}{2}$ **do**    //Crossover operator of mayfly algorithm2:    Select a male parent male and female parent female based on the fitness function3:    offspring1 and offspring2 are calculated according to Equation (8)    //The second crossover operation4:    Randomly select a male parent male and female parent female5:    j=06:    **while** $j⩽P_{C}\times D$ **do**7:         Randomly select a crossover site $x_{k}^{r}$ from *D* sites8:         The crossover site $x_{k}^{r}$ of the male is assigned to offspring1, and the    remaining sites are assigned to offspring2 the site $x_{k}^{r}$ of the female is    assigned to offspring2, and the remaining sites are assigned to    offspring19:        j=j+110:    **end while**11:**end for**

**Figure 6:**
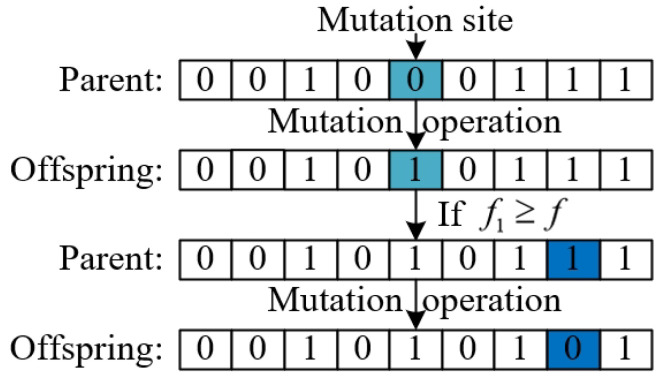
Multi-level mutation operation. Color key: light blue indicate mutation site in the single-level mutation operation; dark blue indicate mutation site in the multi-level mutation operation; white represent sites that remain unchanged (no mutation).

Step2.6: Multi-level mutation operation. In order to prevent the algorithm from falling into a local optimum, the mutation operation of genetic algorithm is introduced. However, the single-level mutation operation, performing the mutation operation only once, has a small search range, and is not easy to jump out of local optimums. Therefore, this paper proposes a multi-level mutation operation; the main steps of the multi-level mutation are displayed in Algorithm 2, which is illustrated in Figure 6.
**Algorithm 2** Multi-level mutation operation**Input:** Male and female populations, num_off, mutation probability $P_{m}$, Threshold of multiple mutation *f*
**output:** offspring populations
1:Create a variable Bests to record the optimal fitness value in all previous iterations2:Create a variable $f_{1}$ to record how many generations of optimal fitness values have not changed3:**for** i=1 to num_off **do**4:    Randomly select a parent    //Single-level mutation operation5:    Genetic algorithm mutation operation based on mutation probability $P_{m}$6:    **if** $f_{1}⩾f$ **then**        //Multi-level mutation operation7:        The individual formed by the single mutation is used as the parent to    perform the mutation operation again.8:    **end if**9:**end for**10:The optimal offspring is selected from the offspring of single-level mutation and multi-level mutation as the offspring of this mutation operation.11:Calculate the optimal fitness value of this round of iteration Bestsloution12:**if** Bestsloution⩽Bests **then**13:    $f_{1}=f_{1}+1$14:**else**15:    Bestsloution=Bests16:    $f_{1}=1$17:**end if**

Step2.7: Elite retention strategies. The updated male population and the first half of the mutated offspring population were merged into a new male population. The individuals of the new male population were sorted by fitness, and the optimal $N_{MA}$ individuals were selected as the next generation of male population. Similarly, the updated female population and the second half of the mutated offspring population are merged, and the fitness values are sorted and screened to obtain the next generation of female population.

Step2.8: Stopping condition judgment for initial parameters. If the stopping condition is satisfied, the global optimal solution is obtained based on the fitness values of all mayfly individuals. Then, the global optimal solution is transformed into the initial weight of the RBF neural network. Afterward, the sample is used to start the neural network training. If the stopping condition is not satisfied, it proceeds to step2.2.

**Figure 7:**
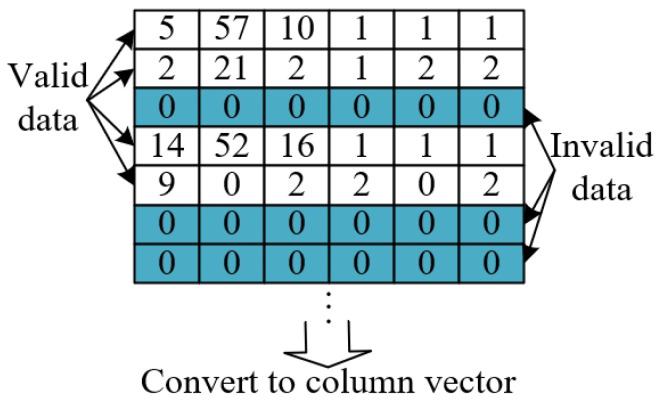
A sample of valid data and invalid data description. Color key: white represent valid data; light blue represent invalid data.

Step3: Selective update strategy. When a sample is provided for the network training at each time, the data in the sample should be classified into valid data and invalid data, as shown in Figure 7. The subsequent step involves integrating the valid data into the neural network, calculating the output value based on the input sample, the center, width of the network, and the weight parameters. Moreover, the MSE value of network prediction for this sample is calculated by comparing with the actual output value. Finally, the weight parameters are updated selectively by means of the gradient descent method. It is noteworthy that only the weight parameters corresponding to the valid data are updated. The main steps of the selective update strategy are displayed in Algorithm  3.
**Algorithm 3** Selective update strategy for RBF neural network**Input:** Input sample $x\in R^{l}$, true output $y\in R^{q}$, centers ${\left\{c_{j}\right\}}_{j=1}^{p}$, widths ${\left\{D_{j}\right\}}_{j=1}^{p}$, weights ${\left\{w_{k,j}\right\}}_{k=1,j=1}^{q,p}$, learning rate η
**output:** Updated weights $\left\{w_{k,j}\right\}$
1:**// Step1: Sample validity check**2:**if** x=0 **then** // (i.e., all elements of x are zero)3:    // *x* is an invalid data4:    // No forward computation or parameter update5:    // Keep weights unchanged6:    **return** $\left\{w_{k,j}\right\}$ unchanged7:**else**8:    // *x* is a valid data9:    // Proceed to forward propagation and selective update 10:    **// Step2: Forward pass and error computation**11:    **for** j=1 to *p* **do**12:        Compute RBF activation: $\phi_{j}\left(x\right)=\exp\left(-\frac{∥x-c_{j}{∥}^{2}}{2D_{j}^{2}}\right)$13:    **end for**14:    **for** k=1 to *q* **do**15:        Compute output: $f_{k}\left(x\right)=\sum_{j=1}^{p}w_{k,j}\cdot \phi_{j}\left(x\right)$16:        Compute error: $e_{k}=f_{k}\left(x\right)-y_{k}$17:    **end for** 18:    **// Step3: Selective gradient update**19:    **for** k=1 to *q* **do**20:        **for** j=1 to *p* **do**21:           Compute gradient: $\frac{\partial L}{\partial w_{k,j}}=e_{k}\cdot \phi_{j}\left(x\right)$22:           Update weight: $w_{k,j}=w_{k,j}-\eta \cdot \frac{\partial L}{\partial w_{k,j}}$23:        **end for**24:    **end for**25:    **return** Updated weights $\left\{w_{k,j}\right\}$26:**end if**

Step4: Stopping condition judgment for network training. If the stopping condition is satisfied, the neural network has completed the training process. Then, the test samples are used to test the performance of the neural network. After that, the real-time information of the AGV system is put into the RBF neural network to predict the task travel time. When the neural network is used to predict, only the valid data is adopted to calculate the MSE value and the prediction travel time. If the stopping condition is not satisfied, the algorithm goes to Step3 to continue the iterative optimization process.

Although the detailed algorithmic steps and flowchart in Figure 4 provide a comprehensive explanation, the full process of the IMA-SURBF algorithm remains computationally intricate. Therefore, to enhance readability and offer a structured overview, Table 2 summarizes the key steps, hyperparameters, outputs, and computational complexity across each phase of the algorithm.

## 4. Experiment

### 4.1. Simulation Model

**Figure 8:**
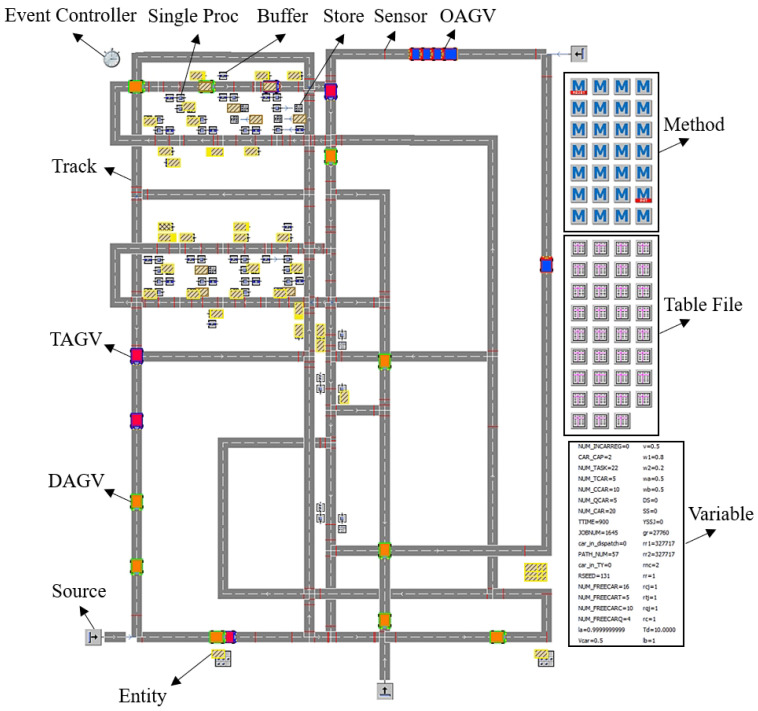
The simulation interface.

To validate the effectiveness of the T3P method in this paper, simulation software Tecnomatix Plant Simulation 15.0 is used to create a simulation model for a logistics system of an assembly line. The simulation interface is shown in Figure 8. AGV, material, and guide path are represented by Transporter, Entity, and Track objects, respectively. The input buffers, work stations, and output buffers are represented by Buffer, SingleProc, and Store objects, respectively. AGVs are monitored and controlled by Sensor objects attached to the Track object. Task dispatching methods are developed by creating Simtalk programs in the Method object. Variables and important data in the system are recorded by using Variable and TableFile objects, respectively. According to the actual scenario, the production cycle of the heterogeneous AGV system is 15 minutes, the load of each multi-load AGV is 2, and the average speed of the AGVs is 0.5 m/s.

### 4.2. Data Preparation

This paper mainly focuses on the simulation system for dispatching heterogeneous AGVs. Travel-influencing factors are introduced as variables into the simulation system to determine the task travel time. Initially, the system state at a certain dispatching moment is randomly generated to simulate various situations encountered by task dispatching. Then, variable data is recorded in the system state area. Thirdly, the simulation data is used to calculate the task travel time for the system task under these conditions.

To comprehensively capture the impact of task state and traffic flow on task travel time, the experiments are conducted 11,000 times, resulting in 11,000 sets of experimental data. Each set includes data about the system variables and the travel time of each task. The first 10,000 sets are used as training samples for the neural network, while the remaining 1000 sets are used as test samples to test the accuracy of T3P model. Additionally, the data is normalized to mitigate potential prediction errors stemming from significant differences in the magnitude of the input/output data and to expedite the training process.

### 4.3. Experimental Results and Discussion

Utilizing the aforementioned simulation model and historical sample data, three distinct experiments are designed: model design experiment, model initialization experiment of IMA-SURBF, and task-dispatching experiment based on IMA-SURBF. Firstly, the model design experiment is used to verify the accuracy of the network model, along with the effectiveness of the selective update strategy. Secondly, the model initialization experiment of IMA-SURBF is used to test the effectiveness and superiority of the IMA algorithm.

#### 4.3.1. Model Design Experiment

Conventional travel time prediction methods are classified into data-driven and mathematical modeling-based methods based on different prediction principles. The time series analysis method is a typical representative of data-driven methods. In this study, both the conventional BP neural network [37] and the RBF neural network, which are commonly used methods in neural networks, along with time series analysis methods [44] and mathematical modeling-based methods [45], are adopted for comparison experiments. The mean absolute percent error (MAPE), mean absolute error (MAE), and mean square error (MSE) are used to analyze and compare the simulation results of the test samples.

The experimental results of the four prediction methods are presented in Table 3. Compared with the time series analysis methods, mathematical modeling-based methods, and BP neural network method, the RBF neural network method exhibits superior performance in travel time prediction (T3P). Boasting the smallest MAPE, MAE, and MSE, it proves to be more accurate for travel time prediction. Due to the random generation of samples with little correlation, the adaptability of the time series analysis method is low. The mathematical modeling-based method cannot fully express the state of the AGV system, and the BP algorithm, which utilizes a gradient descent approach, is more prone to overfitting and slower convergence, especially when handling complex datasets with high variance. Hence, the RBF neural network method is deemed most suitable for T3P in this paper.

Due to the invalid data contained in the historical sample data, this paper adopts the selective update strategy and filters out the invalid data during the neural network test, so as to improve the efficiency of the RBF neural network and to ensure the prediction accuracy. To verify the effectiveness of the proposed method, the RBF neural network with selective update strategy (SURBF) is compared with the conventional RBF. Moreover, to ensure the fair comparison, the key parameters of the neural network are kept the same. The number of nodes in the input layer, hidden layer, and output layer is 228, 342, and 38, respectively, and the initial weight of the neural network, center, and width of the hidden layer are the same.

Table 4 shows the test results of the computational speed of SURBF and conventional RBF. As SURBF filters out the invalid data in the historical sample data, only the valid data is used for RBF training and testing, reducing the calculation amount of RBF network. As shown in Table 3, SURBF has higher efficiency, reducing the training time by 61.19% and the testing time by 38.46% compared with the conventional RBF. It is noteworthy that the training time of the two methods is much longer than the test time. It is because more sample data is used in the RBF training process and a lot of parameters need to update.

**Figure 9:**
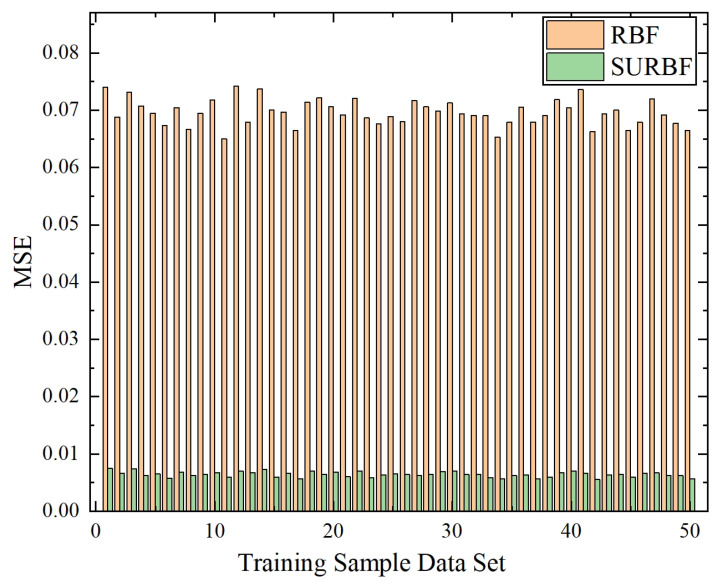
MSE of RBF and SURBF training samples. The MSE values range from 0.005526 to 0.074162 and are calculated on training data.

10,000 sets of sample data are used in the RBF training process to compare the performance of SURBF and conventional RBF networks. The MSE value is collected every 200 sets of training sample data, totaling 50 sets of MSE. Thus, each set of MSE expresses the degree of difference between the predicted value and the actual value based on the 200 sets of training sample data. As shown in Figure 9, the *x*-axis represents the number of training batches (ranging from 1 to 50, each corresponding to 200 training samples), and the *y*-axis denotes the mean squared error (MSE), which is a unitless metric representing the average squared difference between predicted and actual values. The MSE values range from 0.005526 to 0.074162 during training. Compared with the conventional RBF network, the prediction error of the SURBF model is smaller, reducing by 90.77% on average. Due to the different sample data, the MSE value fluctuates in the training process. However, compared with conventional RBF, SURBF has a smaller fluctuation range, and the prediction accuracy is more stable. It is noteworthy that the sampling strategy with an interval of every 200 sets can reduce the effect of data correlation, and ensure that the selected subset is well represents the entire data set.

**Figure 10:**
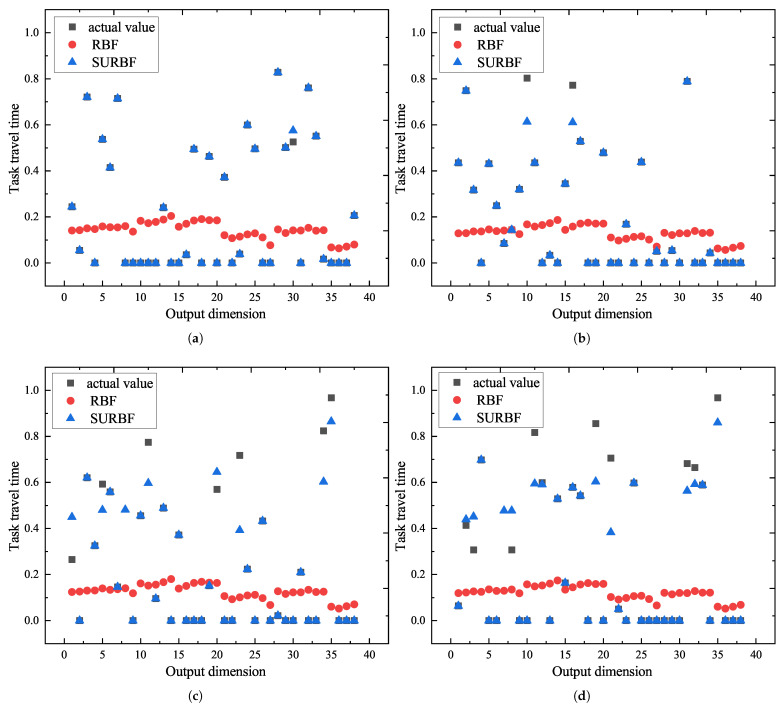
Comparison of actual value and predicted value. (**a**) Test data set 1. (**b**) Test data set 2. (**c**) Test data set 3. (**d**) Test data set 4.

After network training by 10,000 sets of sample data, 1000 sets of test data are used to evaluate the neural network. The performance and generalization ability are evaluated by observing the response and prediction ability of conventional RBF and SURBF to the input data of the test data. In this paper, one set of data is sampled from every 250 sets of these 1000 sets of data, as this sampling method provides a limited and representative comparison across the entire test data set. By analyzing the difference between the predicted value and the actual value of the two RBF neural networks, the performance of the two RBF neural networks can be evaluated more intuitively and comprehensively, and the overall prediction ability of the neural network can be gained. In Figure 10, the x-axis represents the output dimension index, ranging from 1 to 38, corresponding to each sub-task in a test sample. The *y*-axis indicates the normalized values of task travel time, including both the predicted and actual outputs across 38 dimensions. In the 4 sets of test data in Figure 10, the prediction ability of SURBF is significantly better than that of conventional RBF. Despite the intricacy of the problem and the inherent data randomness, occasional errors may occur in limited data points. However, the overall predicted values of the SURBF network is close to the actual values, keeping average error of 0.25%, 1.17%, 6.72%, and 7.85% across the four groups. It verifies the SURBF network has the capability of accurate prediction, robust fitting, and strong generalization. As conventional RBF is susceptible to the influence of invalid data (set to 0 in the dataset), the predicted value varies between 0 and 0.2, further highlighting the necessity of selectively updating the RBF parameters.

To provide a more comprehensive assessment of the prediction accuracy of the traditional RBF neural network and SURBF neural network models, MAPE, MAE, and MSE are used to analyze and compare the simulation results of the test samples. The MAPE, MAE, and MSE values of the SURBF in Table 5 are better than those of the traditional RBF by 89.14%, 87.82%, and 91.2%, respectively. The experimental results show that SURBF achieves the better prediction accuracy.

#### 4.3.2. Model Initialization Experiment

To ensure the population diversity, an improved crossover operation and multi-level mutation operation is introduced to improve the conventional MA. Our improved MA algorithm is termed as IMA for abbreviation. Similarly, the MA with improved crossover operation is abbreviated to CMA, the MA with single-level mutation operation as SMA, and the MA with multi-level mutation operation as MMA. MSE is used as the metrics to compare the performances of the MA invariants.

In the implementation of the improved mayfly algorithm, several key parameters must be configured, including the position variable range, $N_{MA}$, $E_{0}$, $\alpha_{1}$, $\alpha_{2}$, β, *d*, fl, num_off, $P_{C}$, $P_{m}$, and *f*. In reference to the work of Zervoudakis and Tsafarakis (2020), the position variable values are constrained within the range [−1, 1], with an output dimension of 1. However, the proposed IMA-SURBF model features a much higher output dimension of 38, with the number of sample tasks typically ranging from 10 to 20. Unlike other algorithmic parameters that directly influence the evolutionary search dynamics, the position variable range primarily determines the numerical resolution of the search space and does not strongly interact with the internal behavioral mechanisms of the algorithm. For this reason, it was not included in the subsequent parameter sensitivity analysis. Instead, its effect was evaluated in the context of overall model performance in later experiments. Based on these results, the value range of [−1, 1] was adopted as it demonstrated better generalization and prediction accuracy in the final model configuration. In contrast, the remaining parameters lack clear guidance from existing studies due to variations in problem formulation and model structure. Therefore, a comprehensive parameter sensitivity analysis was conducted using mean squared error (MSE) as the primary evaluation metric. A one-factor-at-a-time (OFAT) approach was employed, where each parameter was varied within a predefined range while others were held constant at default values (Table 6).

**Figure 11:**
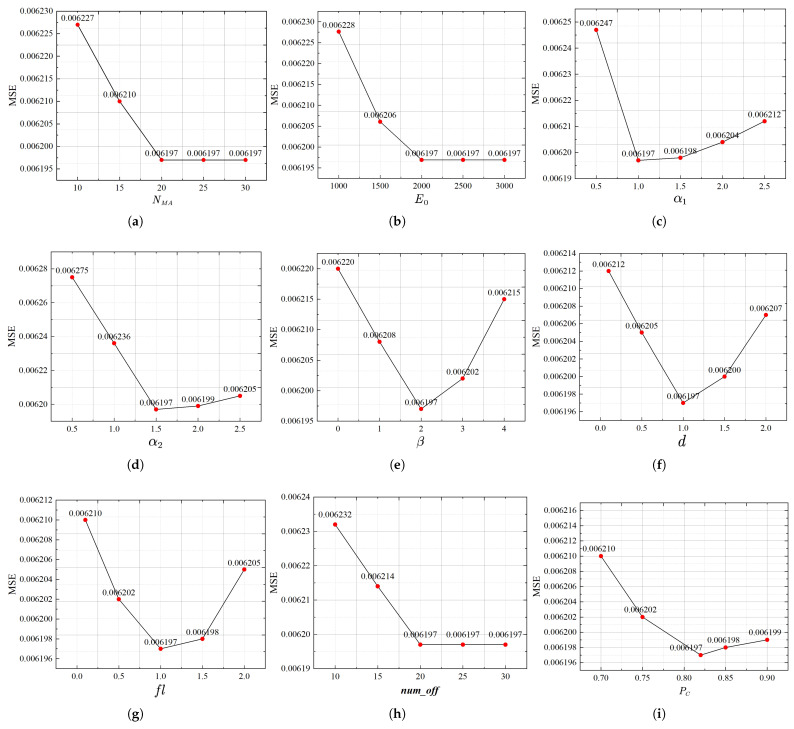

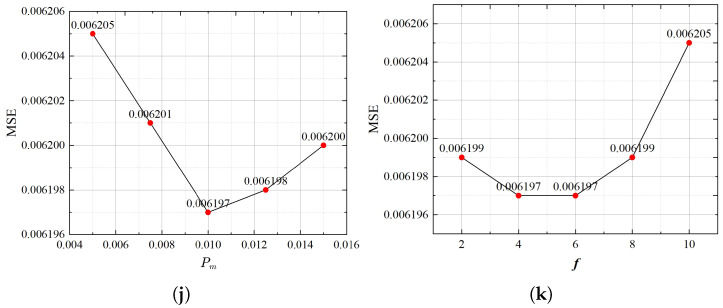
MSE-based sensitivity analysis of IMA parameters. (**a**) Parameter sensitivity analysis of $N_{MA}$. (**b**) Parameter sensitivity analysis of $E_{0}$. (**c**) Parameter sensitivity analysis of $\alpha_{1}$. (**d**) Parameter sensitivity analysis of $\alpha_{2}$. (**e**) Parameter sensitivity analysis of β. (**f**) Parameter sensitivity analysis of *d*. (**g**) Parameter sensitivity analysis of fl. (**h**) Parameter sensitivity analysis of $num_{o}ff$. (**i**) Parameter sensitivity analysis of $P_{C}$. (**j**) Parameter sensitivity analysis of $P_{m}$. (**k**) Parameter sensitivity analysis of *f*.

The experimental results are shown in Figure 11. The maximum iteration count $E_{0}$ significantly influences convergence: MSE decreases notably as $E_{0}$ increases from 1000 to 2000, and stabilizes thereafter, indicating sufficient convergence at the default setting. Population size $N_{MA}$ and offspring number $num_{off}$ affect search diversity and exploration capacity. Both show improved performance as their values increase to moderate levels (e.g., 20), beyond which the MSE remains stable while computational cost increases. Movement-related parameters—including fixed visibility coefficient β, nuptial dance coefficient *d*, and random walk coefficient fl—as well as positive attraction constants $\alpha_{1}$ and $\alpha_{2}$, perform well near their default values. Smaller values may restrict exploration or cause swarm dispersion, while larger values can lead to instability or premature convergence. The crossover probability $P_{C}$, mutation probability $P_{m}$, and threshold of multiple mutation *f* influence exploration strength. Results show that moderate values achieve optimal performance, while overly small or large values slightly affect MSE. The algorithm’s stability under these variations reflects the robustness of its design, particularly the role of elite retention in maintaining high-quality individuals. In summary, although parameter variations are introduced across predefined ranges, prediction errors fluctuate only slightly, demonstrating the strong robustness and adaptability of IMA. The selected default values are well-balanced and capable of delivering stable and reliable prediction results without requiring extensive parameter tuning.

**Figure 12:**
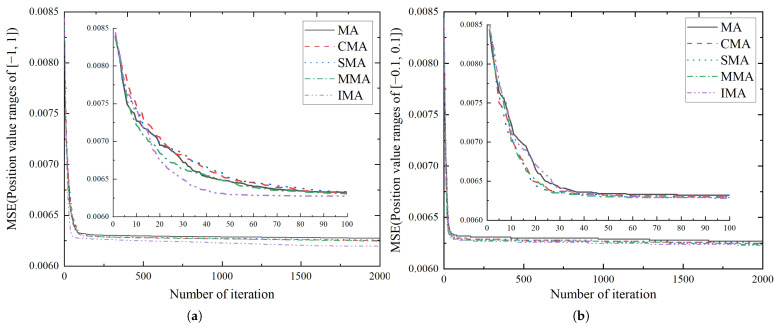
The iteration graph of five MA invariants. (**a**) Position value ranges of [−1, 1]. (**b**) Position value ranges of [−0.1, 0.1].

**Figure 13:**
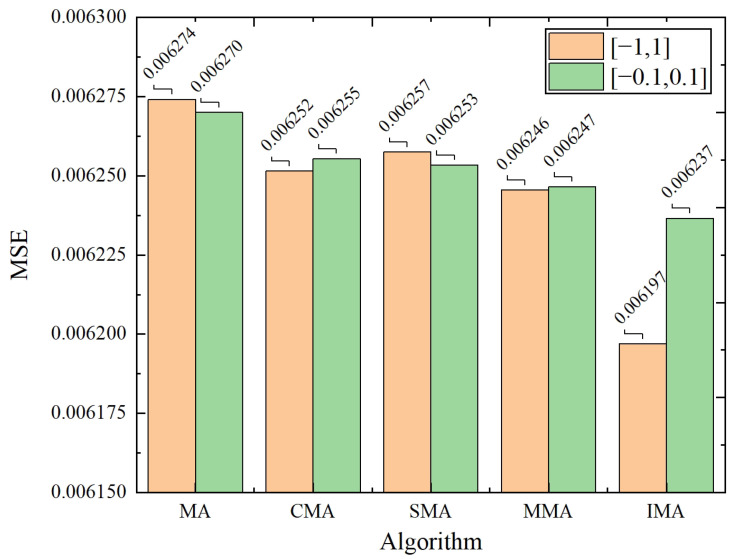
The optimal values of five MA invariants.

Figure 12 and Figure 13 show the iterative process of five MA invariant algorithms and the corresponding optimal values. CMA, SMA, MMA, and IMA exhibit higher convergence speed and optimization ability than conventional MA. Notably, our IMA achieves the best performance. In Figure 12, IMA converges to the optimal value rapidly by the minimal number of iterations on the early stage. The multi-level mutation operation is proven to have superior global optimization ability to the single-level mutation operation. It allows IMA to have better ability to escape from the local optimum, and to search individuals with better fitness. The data comparison in Figure 13 indicates that the optimal value obtained by IMA is over 1.2% higher than that of other mayfly algorithms, highlighting its stronger global optimization ability. It should be noted that the numerical differences are relatively small, and the results are presented primarily for qualitative comparison.

To further demonstrate the advantages of IMA, three commonly-used intelligent optimization algorithms are adopted for the comparison experiments: genetic algorithm (GA) [46], artificial bee colony algorithm (ABC) [47], and particle swarm optimization (PSO) [48]. The parameter settings for each algorithm are listed in Table 7. To observe the influence of the position value on each algorithm, the value ranges are chosen both in the [−1, 1] and [−0.1, 0.1] intervals.

**Figure 14:**
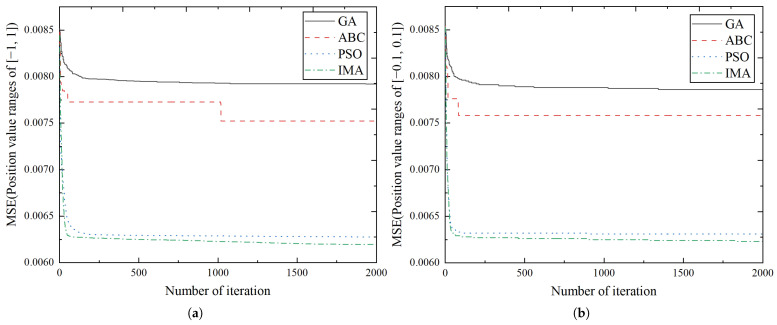
The graph of 2000 iterations of GA, ABC, PSO, and IMA. (**a**) Position value ranges of [−1, 1]. (**b**) Position value ranges of [−0.1, 0.1].

**Figure 15:**
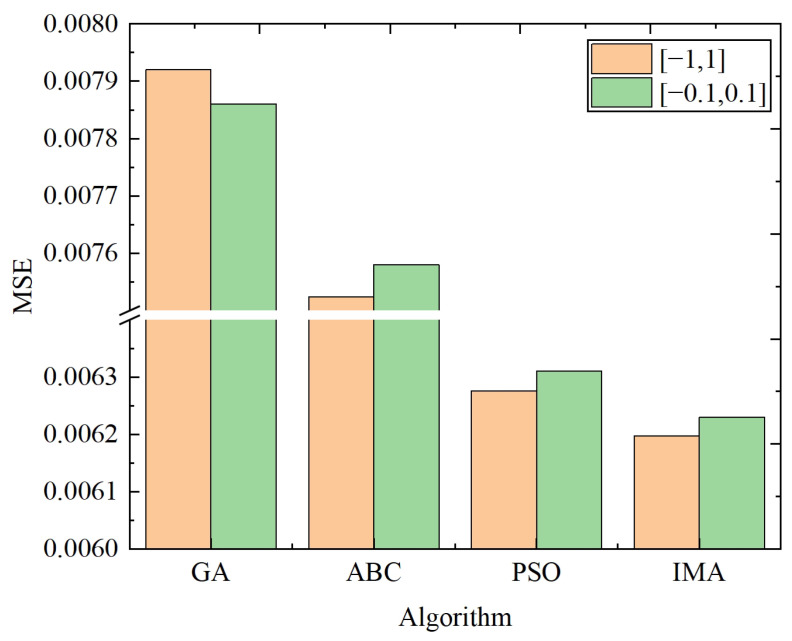
The optimal values of GA, ABC, PSO, and IMA.

Figure 14 and Figure 15 show the iterative process and the optimal values of the GA, ABC, PSO, and IMA, respectively. In Figure 15, IMA obtains a more optimal value compared with GA, ABC, and PSO, i.e., 21.75%, 17.63%, and 1.27% higher than them in the range of [−1, 1], and 20.73%, 17.81%, and 1.26% higher in the range of [−0.1, 0.1]. As the curves shown in Figure 14, IMA not only converges much faster than other algorithms, but also obtains the higher-quality solution for the optimization problem. Further, Figure 14 and Figure 15 show that faster convergence can be attained in the [−0.1, 0.1] value range for all algorithms, but at the cost of accuracy decrease. As the prediction accuracy is prioritized in our model, the [−1, 1] value range is adopted for the position variable in our model.

Since the direct testing method does not involve gradient-based parameter updates, it excludes comparisons of mean squared error (MSE) on training samples. To investigate the influence of each algorithm on the training performance of SURBF in the update test method, MSE values were collected across 10 independently generated training datasets, each containing 1000 samples.

A comparative analysis was then performed among RBF models optimized by different intelligent algorithms to assess the predictive improvement achieved by IMA. As shown in Table 8, all optimization strategies led to significant reductions in MSE compared to the baseline RBF, underscoring the importance of effective parameter initialization in enhancing model performance. Among the tested models, IMA-RBF yielded the lowest average MSE, outperforming other variants such as GA-RBF, PSO-RBF, and ABC-RBF. The corresponding reduction percentages are summarized in Table 9. Specifically, IMA-RBF achieved the highest average improvement rate of 5.17%, while other mayfly algorithm-based variants (CMA-RBF, SMA-RBF, MMA-RBF) exhibited consistent performance gains ranging from 4.41% to 4.55%. In contrast, traditional algorithms such as GA and PSO demonstrated relatively modest improvements of 3.74% and 3.98%, respectively.

These results confirm the superiority of IMA in the context of RBF-based task travel time prediction. The integration of multi-level mutation and adaptive exploration mechanisms within IMA contributes to improved the training efficiency and predictive accuracy of RBF models. Furthermore, this comparative experiment forms an essential component of individual experiments, providing empirical support for the subsequent evaluation of the complete IMA-SURBF framework.

To observe the effect of each optimization algorithm on the performance of SURBF models in the update test approach, 10 groups of training samples in which each group contains 1000 sets of training data are used to calculate the MSE values for these SURBF invariant methods, as shown in Table 10 and Table 11.

Table 10 and Table 11 show that the MSE values of SURBF invariants have been reduced to different extents on these 10 groups of training samples. IMA-SURBF obtains the maximum reduction (7.56%) for the max MSE value, while ABC-SURBF has the minimum reduction (0.14%) for the min MSE value. Moreover, IMA-SURBF also achieves the maximum reduction (2.47%) for the average MSE value. The results indicate that optimizing the initial parameters by using IMA can effectively reduce the training error of SURBF model.

**Figure 16:**
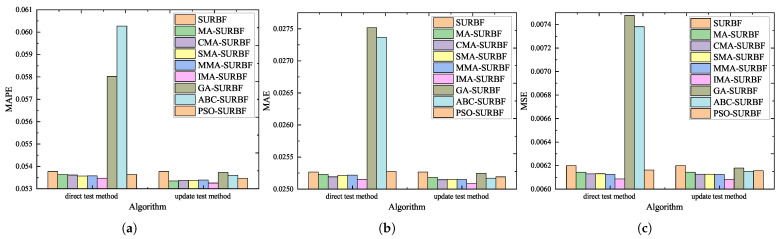
Error analysis of SURBF invariants. (**a**) MAPE comparison. (**b**) MAE comparison. (**c**) MSE comparison.

After that, SURBF invariants are tested by using 1000 sets of test samples. MAPE, MAE, and MSE metrics are used to analyze the prediction errors, as shown in Figure 16. MA-SURBF, CMA-SURBF, SMA-SURBF, MMA-SURBF, and IMA-SURBF exhibit better performance than SURBF in both the direct test and update test approaches. However, the errors of GA-SURBF and ABC-SURBF are obviously larger than SURBF for the direct test approach.

Two probable reasons include that the direct test approach cannot use the selective update strategy to update the RBF network parameters, and that GA and ABC algorithms are not suitable to entirely replace the gradient descent method for parameter updating. Nevertheless, all SURBF invariants have better performance than conventional SURBF for the update test approach. In this framework, GA, ABC, and PSO can also help to optimize the initial parameters of the SURBF network. Moreover, the update test approach outperforms the direct test approach for IMA-SURBF invariants in MAPE and MAE metrics. It demonstrates that the approach of optimizing the initial parameters of the SURBF network before network training and selectively updating the partial network parameters relevant to the input/output channels that have valid training data is effective for the T3P problem.

In summary, the experiments conducted in this study evaluate the effectiveness of the proposed IMA-SURBF model for T3P in multi-AGV systems. To be specific, SURBF demonstrated superior prediction accuracy compared to conventional RBF, time series analysis, and mathematical modeling-based methods, achieving significant reductions in MAPE, MAE, and MSE. SURBF also showed enhanced computational efficiency, reducing training time. Additionally, the initialization of model parameters using the improved mayfly algorithm significantly improves the training efficiency and predictive accuracy of RBF and SURBF models. Compared with other intelligent optimization algorithms, IMA exhibited superior convergence speed, global optimization ability, and robustness. These results highlight the advantages of the IMA-SURBF framework in addressing the complexities of T3P, offering accurate, efficient, and reliable solutions for heterogeneous AGV task scheduling.

#### 4.3.3. Comparison with Deep Learning-Based Models

To assess the performance and practical applicability of the proposed IMA-SURBF framework, it is compared against three commonly used deep learning models for time series forecasting: long short-term memory (LSTM) [49], Gated Recurrent Unit (GRU) [50], and a Transformer-based model constructed with reference to TFT [51] and MCT-TTE [52]. All models are trained and evaluated on the same dataset using identical input features and preprocessing methods. In order to ensure fair comparison, each deep learning model is tuned to its best configuration before final evaluation. A grid search is performed to optimize key hyperparameters, including the learning rate, batch size, hidden-layer number, hidden-layer size, and dropout rate. Moreover, two strategies of early stopping and adaptive learning rate adjustment are employed to prevent overfitting, by automatically terminating the training process when the validation performance plateaus. Four key metrics, i.e., MAPE, MAE, MSE, and test time, are considered in the evaluation process, where test time refers to the total inference time of processing 1000 test samples.

As summarized in Table 12, IMA-SURBF consistently achieves the best accuracy across all error metrics. Compared with LSTM and GRU, IMA-SURBF reduces MAE by over 50%, and improves MAPE and MSE by more than 19%. The Transformer-based model yields lower-than-expected results across all metrics, suggesting that its architecture may not be well-aligned with the relatively short and structured input sequences common in AGV task travel time prediction. Regarding inference efficiency, IMA-SURBF completes the test set over seven times faster than LSTM, more than eight times faster than GRU, and over twenty times faster than the Transformer model. These differences highlight the computational advantages of the proposed method, especially in time-sensitive industrial scenarios. Furthermore, IMA-SURBF provides better interpretability and transparency than black-box deep learning models, owing to its selective update mechanism and simpler structure.

In summary, IMA-SURBF achieves an effective balance of prediction accuracy, inference speed, and model interpretability, making it well-suited for real-time deployment in heterogeneous AGV systems.

## 5. Conclusions

Task travel time is a critical factor in the task dispatching of heterogeneous AGV systems. However, accurate prediction is challenging due to the correlation between individual tasks and the dynamic changes in the model’s input and output dimensions. To improve the prediction accuracy of AGV task travel time, a T3P model is constructed by taking travel-influencing factors as input and the task travel time as output of an RBF neural network, while the input/output dimension is adjusted dynamically in terms of the current task number. IMA is used to optimize the initial weights of the RBF neural network, in order to promote the population diversity and the global searching ability of the mayfly algorithm. The experimental results show that our IMA-SURBF method predicts the task travel time more accurately, compared with other SURBF invariants, the time series analysis and mathematical modeling-based method significantly.

Further research will be carried out to address the following limitations. First, due to the lack of access to on-site operational data from enterprises, all historical samples used in this study were generated through simulation. Although the simulation environment can approximate various operational conditions, it cannot fully capture the complexity of real-world scenarios, such as sensor noise, AGV path anomalies, and unexpected task interruptions. Therefore, future work will focus on collecting real-world AGV operational data to retrain and validate the IMA-SURBF model in practical industrial settings. In particular, extensive testing will be carried out within integrated multi-line manufacturing systems to comprehensively assess the model’s generalizability and robustness in large-scale, dynamic AGV task-dispatching scenarios. Second, although the IMA-SURBF model incorporates multi-source feature fusion and multi-level optimization mechanisms, the experimental results demonstrate that it achieves high prediction accuracy with relatively low inference time, indicating its potential for near real-time deployment. In future work, we plan to further enhance computational efficiency through GPU-based parallelization and algorithmic streamlining. Owing to the modular and parallelizable nature of the proposed framework, it is expected to scale effectively in larger industrial systems while maintaining high performance.

## Figures and Tables

**Table 1:** Relevant research works to travel time prediction.

References	Field	Algorithm	Influencing Factor	City	Data Source
[18]	Road network	TRIP	Local conditions	Seattle	GPS
[19]	Bus	Linear regression	Traffic conditions	Shanghai	GPS and RFID
[20]	Urban arterials	Multiple linear regression	Road environment and flow characteristics	Low-income country	Moving observer method
[21]	Bus	BMAR Model	Spatial–temporal factors and unexpected events	Klang Valley	AVL system
[22]	Commercial vehicles	Discrete and continuous combined analysis	Multiple factors	China	The trajectory data set
[30]	Roadway	Linear regression and ANN	Bus GPS data	Chennai	GPS, Wi-Fi and Bluetooth
[31]	Urban roads	TTDP-GAN	Traffic data collected	Medium-sized city	License plate recognition data
[32]	Bus	Machine learning	Bus-stop arrival time data	Istanbul	Automatic vehicle location data
[33]	Signalized corridor	ANN-Bayes-based	Interrupted traffic flows	State of Ohio	Probe data
[34]	Bus	Hybrid neural network	Historical bus travel data	Germany	HOCHBAHN bus company
[35]	Bus	BEDDNN	Uncertainty	L city	GPS position and time

Note: TRIP—for travel time reliability inference and prediction, GPS—global positioning systems; RFID—radio frequency identification; BMAR—Burr mixture autoregressive; AVL—automatic vehicle location; ANN—artificial neural network; TTDP-GAN—the travel time distribution prediction-generative adversarial network; BEDDNN—Bayesian encoder–decoder deep neural network.

**Table 2:** Summary of the IMA-SURBF algorithm.

Phase	Step Description	Key Parameters	Output	Computational Complexity (Order)
1. Initialization	K-means clustering to initialize RBF centers and widths	*p*: number of centers; *n*: input dimension; $E_{kmeans}$: max iterations of K-means	Centers $C_{j}$, Widths $D_{j}$	$O(p\cdot n\cdot E_{kmeans})$
2. Weight Optimization via IMA	Step2.1: Generate initial mayfly population	$N_{MA}$, D=p·q, $E_{0}$	Population positions and velocities	$O(N_{MA}\cdot D)$
	Step2.2: Evaluate fitness using MSE from trained RBF	Sample size *N*	Fitness values for each mayfly	$O(N_{MA}\cdot N\cdot p\cdot q)$
	Step2.3: Update local/global bests $p^{r}$, $p^{g}$	—	Updated bests	$O(N_{MA})$
	Step2.4: Position & velocity update for male/female	$\alpha_{1}$, $\alpha_{2}$, β, γ, fl	Updated populations	$O(N_{MA}\cdot D)$
	Step2.5: Improved crossover operation	$P_{c}$, $num_{off}$	New offspring	$O(num_{off}\cdot D)$
	Step2.6: Multi-level mutation operation	$P_{m}$, *f*	Mutated offspring	$O(num_{off}\cdot D)$
	Step2.7: Elite selection and next generation update	—	New populations	$O(N_{MA}\cdot \log N_{MA})$
	Step2.8: Check stopping condition	$E_{0}$, fitness threshold	Stopping criterion	—
3. Selective Update	Gradient descent update for valid samples only	valid data	Updated weights $w_{k,j}$	$O(N_{\mathrm{valid}}\cdot p\cdot q)$
4. Termination	Check training termination	Max iterations or tolerance	Trained RBF model	—

**Table 3:** Comparison of test set errors for three prediction methods.

Method	MAPE	MAE	MSE
Time series analysis	1.855593124	0.213577099	0.084671574
Mathematical modeling-based	0.822642668	0.213019803	0. 076543313
BP	0.421496931	0.209750225	0.725667623
RBF	0.494787295	0.207277712	0.070234889

**Table 4:** The computational speed comparison between SURBF and RBF.

Method	Training Time (s)	Testing Time (s)
RBF	38,340	26
SURBF	14,880	16

**Table 5:** Comparison of RBF and SURBFP test set errors.

Method	MAPE	MAE	MSE
RBF	0.494787295	0.207277712	0.070234889
SURBF	0.053767268	0.025266458	0.006199791

**Table 6:** Parameter ranges and default values for IMA sensitivity analysis.

Parameter	Predefined Range	Default Value
$N_{MA}$	10, 15, 20, 25, 30	20
$E_{0}$	1000, 1500, 2000, 2500, 3000	2000
$\alpha_{1}$	0.5, 1.0, 1.5, 2.0, 2.5	1.0
$\alpha_{2}$	0.5, 1.0, 1.5, 2.0, 2.5	1.5
β	0, 1.0, 2.0, 3.0, 4.0	2.0
*d*	0.1, 0.5, 1.0, 1.5, 2.0	1.0
fl	0.1, 0.5, 1.0, 1.5, 2.0	1.0
num_off	10, 15, 20, 25, 30	20
$P_{C}$	0.75, 0.8, 0.82, 085, 0.9	0.82
$P_{m}$	0.005, 0.0075, 0.01, 0.0125, 0.015	0.01
*f*	2, 4, 6, 8, 10	6

**Table 7:** Parameter settings for comparative algorithms.

Algorithm	Parameter settings
GA	The population size is 40, the number of iterations is 2000, the selection operator is roulette wheel selection, the crossover probability is 0.85, and the mutation probability is 0.01.
ABC	The population size is 40, the number of iterations is 2000, "limit" is 100.
PSO	The population size is 40, the number of iterations is 2000, cognitive coefficient $c_{1}$ is 2, and social coefficient $c_{2}$ is 2.

**Table 8:** MSE comparison of RBF models optimized by different intelligent algorithms on training samples.

NO	RBF	MA-RBF	CMA-RBF	SMA-RBF	MMA-RBF	IMA-RBF	GA-RBF	ABC-RBF	PSO-RBF
1	0.282746	0.275187	0.273243	0.273830	0.273659	0.271069	0.278275	0.276315	0.276127
2	0.302867	0.289166	0.288402	0.287381	0.287650	0.284690	0.289963	0.292934	0.289961
3	0.287371	0.257434	0.257334	0.257220	0.256775	0.255697	0.258252	0.259842	0.257985
4	0.303871	0.299139	0.297374	0.298708	0.297429	0.296256	0.299711	0.299790	0.298976
5	0.294652	0.288672	0.287497	0.288166	0.287547	0.285780	0.289293	0.291149	0.289059
6	0.311155	0.297169	0.295781	0.296652	0.295829	0.293417	0.298192	0.300520	0.297315
7	0.280906	0.265788	0.264266	0.264719	0.263685	0.263454	0.266524	0.268134	0.265687
8	0.311237	0.308399	0.307772	0.307532	0.307181	0.305243	0.310116	0.307512	0.309693
9	0.260777	0.250635	0.250566	0.250679	0.250013	0.248058	0.251948	0.255918	0.251018
10	0.270150	0.255241	0.254640	0.254151	0.255007	0.253064	0.256151	0.257842	0.255566
Average	0.290573	0.278683	0.277687	0.277904	0.277478	0.275673	0.279843	0.280996	0.279139

**Table 9:** MSE reduction percentage of optimized RBF models compared with the baseline RBF.

NO	MA-RBF	CMA-RBF	SMA-RBF	MMA-RBF	IMA-RBF	GA-RBF	ABC-RBF	PSO-RBF
Max	10.42%	10.45%	10.49%	10.65%	11.02%	10.13%	9.58%	10.23%
Min	0.91%	1.11%	1.19%	1.30%	1.93%	0.36%	1.19%	0.50%
Average	4.14%	4.48%	4.41%	4.55%	5.17%	3.74%	3.32%	3.98%

**Table 10:** MSE comparison of SURBF invariants for training samples.

NO	SURBF	MA-SURBF	CMA-SURBF	SMA-SURBF	MMA-SURBF	IMA-SURBF (Ours)	GA-SURBF	ABC-SURBF	PSO-SURBF
1	0.006857	0.006394	0.006380	0.006391	0.006378	0.006338	0.006603	0.006585	0.006405
2	0.006392	0.006335	0.006321	0.006333	0.006316	0.006272	0.006357	0.006379	0.006358
3	0.006569	0.006521	0.006508	0.006509	0.006507	0.006452	0.006534	0.006560	0.006528
4	0.006528	0.006461	0.006450	0.006456	0.006450	0.006412	0.006482	0.006509	0.006488
5	0.006364	0.006292	0.006288	0.006292	0.006288	0.0062330	0.006310	0.006347	0.006321
6	0.006590	0.006528	0.006522	0.006522	0.006514	0.006474	0.006542	0.006577	0.006550
7	0.006117	0.006052	0.006041	0.006053	0.006039	0.006005	0.006075	0.006096	0.006074
8	0.006343	0.006278	0.006273	0.006286	0.006272	0.006223	0.006295	0.006331	0.006306
9	0.006173	0.006115	0.006108	0.006117	0.006101	0.006059	0.006136	0.006161	0.006133
10	0.006295	0.006229	0.006222	0.006221	0.006215	0.006174	0.006237	0.006272	0.006250
Average	0.006423	0.006321	0.006311	0.006318	0.006308	0.006264	0.006382	0.006382	0.006357

**Table 11:** MSE reduction percentage of SURBF invariants compared with conventional SURBF.

Algorithm	MA-SURBF	CMA-SURBF	SMA-SURBF	MMA-SURBF	IMA-SURBF (Ours)	GA-SURBF	ABC-SURBF	PSO-SURBF
Max	6.75%	6.95%	6.80%	6.99%	7.56v	3.70%	3.97%	6.60%
Min	0.73%	0.93%	0.90%	0.94%	1.76%	0.53%	0.14%	0.53%
Average	1.59%	1.74%	1.63%	1.79%	2.47%	1.02%	0.64%	1.27%

**Table 12:** Prediction accuracy and test time comparison between IMA-SURBF and deep learning baselines.

Model	MAPE	MAE	MSE	Test Time (s)
LSTM	0.069851	0.053736	0.007497	123
GRU	0.074795	0.056472	0.007568	138
Transformer	0.092125	0.073438	0.013121	357
IMA-SURBF	0.053209	0.025005	0.006051	16

## Data Availability

No new data were created or analyzed in this study. Data sharing is not applicable to this article.
